# Data on a computationally efficient approximation of part-powder conduction as surface free convection in powder bed fusion process modelling

**DOI:** 10.1016/j.dib.2019.104559

**Published:** 2019-09-23

**Authors:** Wenyou Zhang, Mingming Tong, Noel M. Harrison

**Affiliations:** a Mechanical Engineering, College of Engineering & Informatics, NUI Galway, Ireland; b I-Form Advanced Manufacturing Research Centre, Ireland; c IComp Irish Composites Centre, Ireland; d Ryan Institute for Environmental, Marine and Energy Research, NUI Galway, Ireland

**Keywords:** Powder bed fusion, Process modelling, Part-powder conduction, Surface free convection

## Abstract

This article is related to research article entitled “Resolution, energy and time dependency on layer scaling in finite element modelling of laser beam powder bed fusion additive manufacturing” [1]. This data article presents a computationally efficient approximation of part-powder interface conduction heat transfer, as convection heat transfer, thus eliminating the need for powder elements in the finite element model. The heat loss profile due to part-powder conduction was first characterised for a Ti6Al4V Powder Bed Fusion process. Cooling rate data was obtained for a range of powder in-plane depths. A matching cooling rate profile was obtained from free convection from the part surface, by calibration of the convection coefficient.

**Table:** Specifications Table

Subject	Mechanical Engineering
Specific subject area	Additive Manufacturing/Powder Bed Fusion, Finite Element Modelling
Type of data	Graph, Figure
How data were acquired	Numerical simulation was performed in ABAQUS (Dassault Systems, USA, 2017)
Data format	Raw and analysed
Parameters for data collection	The nodal temperature data was collected from identical nodes (data points) in the ABAQUS heat transfer computational model.
Description of data collection	The simulation was carried out using a software tool ABAQUS. Two sets of heat transfer models were developed: one set of heat conduction model varying powder in plane depth and one set of heat convection model. Each simulation run for the same time of 1 hour. The nodal temperatures were output and plotted in the figure.
Data source location	National University of Ireland, Galway, Ireland.
Data accessibility	Data are available within this article and on Mendeley data: https://doi.org/10.17632/757zr92cg9.3#folder-b21bc92d-d81e-4cc0-9780-632dc68fb31d
Related research article	W. Zhang, M. Tong, N.M. Harrison, Resolution, energy and time dependency on layer scaling in finite element modelling of laser beam powder bed fusion additive manufacturing, Addit. Manuf. 28 (2019) 610–620.https://doi.org/10.1016/j.addma.2019.05.002[1].

**Table:** 

**Value of the data** • The presented data is useful for computationally efficient of powder bed fusion modelling by eliminating powder elements from the finite element model. • This data can be used as a reference for cooling rates for solid parts in powder bed fusion with a range of powder through-thicknesses • The presented cooling rates are valuable data on heat transfer mechanisms of powder bed fusion and can be beneficial for additive manufacturing part designers, build layout designers and equipment operators. • The data includes cooling rates for multiple computational heat transfer models. • The data can be used to inform powder bed fusion build experiments on appropriate data sampling (frequency and location) for heat transfer measurements.

## 1 Data

**Fig. 1:**
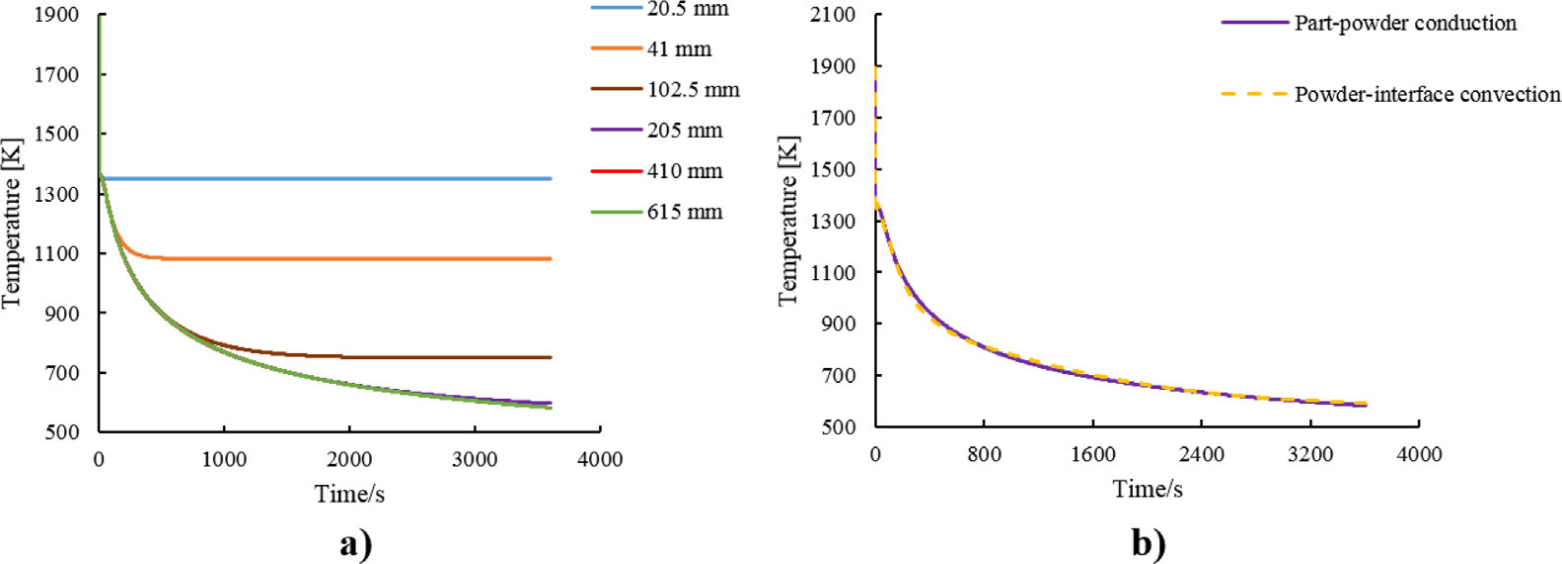
Part-powder thermal loss: a) Ti6Al4V powder in plane depth verification during the part-powder conduction. b) The cooling curves of part-powder conduction and powder-interface convection.

One of the primary heat transfer mechanisms in Laser Beam Powder Bed Fusion (PBF-LB) is heat loss from the printed part to the surrounding non-solidified powder. Powder in-plane depth (i.e. thickness of surrounding powder) has a significant influence on both the cooling rate as well as the steady-state temperature. To date, most finite element simulations of PBF-LB component manufacturing include the powder elements in the finite element model, and are limited to small volumes, due to computational cost. Data is presented for equivalent cooling of the solid part via convection from the part surface, without any powder elements. Fig. 1a indicates the cooling rate of part-powder conduction at the powder interface with different powder in plane depths. In all cases, the temperature initially decreases exponentially, followed by steady state. For this model configuration, the greater the powder in-plane depth, the lower of the final temperature: 600 K and 1350 K interface temperature for powder in-plane depths of 20.5 mm and 410 mm, respectively. The heat loss rate converges at 205 mm powder in-plane depth, i.e. larger powder-in plane depths has minimal influence on surface temperature.

The time-dependant film coefficient defined for the powder-interface convection model was calibrated to fit the resulting free surface convection cooling curve to the 205 mm powder in plane depth cooling profile (Fig. 1a). The resulting fit of the convection-based approximation of the conduction-based cooling mechanism is shown in Fig. 1b.

This time-dependant surface convection definition for Ti6Al4V could be employed for every layer, in lieu of including (heat conductive) powder elements [1].

## 2 Experimental design, materials, and methods

Erik R. et al. [2] showed the importance of incorporating loose powder along with the solid part in FE modelling of LPBF, due to the need to capture the solid part to powder conduction heat transfer. The heat loss profile due to part-powder conduction was first characterised, by simulating a single layer consisting of a solid region (exposed to a heat flux) and a powder region. The powder region experiences heating due to conduction from the solid part only. In practice the total heat loss due to this mechanism will depend on the powder in-plane depth, i.e. how densely packed the build volume is with parts, and how close the parts are to the chamber walls. Therefore, a range of powder in-plane depths were simulated (20.5 mm, 41 mm, 102.5 mm, 205 mm, 410 mm, 615 mm) for a 20.5 mm wide solid part, and the temperature at the part-powder interface was monitored.

A new model was prepared with the powder removed and powder–interface convection was defined at the part surface (formerly the powder-part interface). The convection coefficient was calibrated to produce the same cooling profile as the converged cooling profile from (Fig. 1b). Material definitions for Ti6Al4V powder were assumed to be simply related to the solid material properties via powder bulk porosity *p* [3]:

(1) $$\;\rho_{p}=\rho_{s}\left(1-p\right)$$

(2) $$k_{p}=k_{s}\left(1-p\right)$$

A powder porosity *p* of 40.5% was determined based on published powder (2.63 g/cm^3^ [4]) and solid (4.42 g/cm^3^ [5]) Ti6Al4V. Therefore, a porosity *p* of 40.5%, thus allowing $k_{p}$ to be determined based on the temperature dependant$\;k_{\text{s}}$. The thermal conductivity of powder Ti6Al4V was assumed to equal solid Ti6Al4V above the melt point of 1893 K. The remaining powder thermal properties of specific heat of powders were considered to equal solid properties [6], [7], [8].

Active layer convection and radiation occurs at the top surface of the (newly added) active layer during the cooling step time and before becoming inactive after next layer is added. The surface heat loss due to the active layer convection can be expressed by Newton's law of cooling:

(3) $$q_{conv}=h\left(T_{s}-T_{r}\right)$$

where *q*_*conv*_ is the heat flux due to convection, *h* is the heat transfer coefficient of convection [9], *T*_*s*_ is the surface temperature and *T*_*r*_is the chamber temperature [3]. Here, the chamber temperature was 293 K, which was the same with the initial temperature of powder and the characteristic heat transfer coefficient *h* was found to be 12.7 W/m^2^ K [10] with assuming that *h* is independent of temperature. The heat dispersion due to the active layer radiation can be defined by Stefan-Boltzmann's law:

(4) $$q_{rad}=\varepsilon \;\sigma \left({T_{s}}^{4}-{T_{r}}^{4}\right)$$

where ε is the emissivity coefficient, *σ* is the Stefan-Boltzmann constant, *T*_*s*_ is the surface temperature and *T*_*r*_is the ambient temperature. Here emissivity of the active layer surface was set as 0.35 [10] and the Stephan's constant was defined as σ = 5.669 × 10^−8^ W/m^2^ K^4^ [11]. Between two subsequent layers, a representative cooling step time of 6 s was used to model the time needed by the coater to spread the new powder layer.
